# Desbuquois dysplasia and cardiovascular complications: a retrospective cohort study

**DOI:** 10.1007/s00431-025-06231-4

**Published:** 2025-06-03

**Authors:** Musa Öztürk, Merve Tanrısever Türk, Gizem Ürel Demir, Gülen Eda Ütine, İlker Ertuğrul, Ebru Aypar, Tevfik Karagöz, Dursun Alehan, Pelin Özlem Şimşek Kiper, Hayrettin Hakan Aykan

**Affiliations:** 1 Department of Pediatric Cardiology, Faculty of Medicine, Hacettepe University, Ankara, Turkey; 2 Department of Pediatric Genetics, Faculty of Medicine, Hacettepe University, Ankara, Turkey; 3 Life Support Center, Hacettepe University, Ankara, Türkiye

**Keywords:** Desbuquois dysplasia, Aortic root dilatation, Aortopathy, Cardiovascular manifestations

## Abstract

Desbuquois dysplasia (DBQD) is a rare autosomal recessive chondrodysplasia characterized by distinct skeletal abnormalities and multisystem involvement. Cardiac manifestations, such as aortic root dilatation and mitral valve prolapse, have also been reported, likely due to impaired proteoglycan production. This study aims to enhance the understanding of clinical management and cardiac implications in patients with DBQD, contributing to the broader knowledge of this rare condition. This research was conducted at Hacettepe University İhsan Doğramacı Children’s Hospital, a tertiary reference center for all pediatric subspecialties. A single-center, descriptive, retrospective cohort study was performed. Demographic characteristics, genetic mutations, echocardiographic findings, and measurements of patients with Desbuquois dysplasia were documented. A total of nine patients, including five females (55%) were included in the study. The median age of the patients was 11 years (range 3.6–23.6 years), the median body weight was 15 kg (6–64 kg), and the median height was 94 cm (63–130 cm). The median follow-up period was 7.7 years (range 2.9–15.4 years). All patients had homozygous or compound heterozygous pathogenic variants in the CANT1 gene. The most common cardiac findings included mitral valve prolapse (seven patients, 77%), ascending aortic dilatation (seven patients, 77%), aortic root enlargement (six patients, 66%), small atrial septal defect (ASD) (five patients, 55%), bicuspid aortic valve (two patients, 22%), and ventricular septal defect (VSD) (one patient, 11%). Additionally, coronary-cameral fistula, a rare finding in the general population, was observed in one patient. The median individual *Z* scores for the sinus valsalva (SVS) in patients with aortic dilatation were 4.9 (range 2.7–7.5), while the median *Z* score in the ascending aorta was 5 (range 2.3–8.5). *Conclusion*: Aortic root and ascending aorta dilatation as well as mitral valve prolapse are frequently observed in patients with DBQD. ASD, VSD, and bicuspid aorta are less common. Aortopathy develops early and can progress to a severe stage. Early detection of cardiac abnormalities and timely initiation of medical treatment may significantly improve the long-term prognosis of the disease.

**Table:** 

**What is Known:** *• Desbuquois dysplasia (DBQD) is a rare autosomal recessive chondrodysplasia characterized by distinct skeletal abnormalities and multisystem involvement. Cardiac manifestations, such as aortic root dilatation and mitral valve prolapse, have also been reported, likely due to impaired proteoglycan production.*	
**What is New:** *• The most frequently observed findings include aortic root and ascending aortic dilatation as well as mitral valve prolapse. Aortopathy develops early and can progress to severe disease. Early detection of cardiac abnormalities and timely initiation of medical treatment may significantly improve long-term prognosis.*

**What is Known:**

*• Desbuquois dysplasia (DBQD) is a rare autosomal recessive chondrodysplasia characterized by distinct skeletal abnormalities and multisystem involvement. Cardiac manifestations, such as aortic root dilatation and mitral valve prolapse, have also been reported, likely due to impaired proteoglycan production.*

**What is New:**

*• The most frequently observed findings include aortic root and ascending aortic dilatation as well as mitral valve prolapse. Aortopathy develops early and can progress to severe disease. Early detection of cardiac abnormalities and timely initiation of medical treatment may significantly improve long-term prognosis.*

## Introduction

Desbuquois dysplasia (DBQD) is a form of chondrodysplasia inherited in an autosomal recessive pattern. It is characterized by severe micromelic dwarfism, joint laxity, progressive scoliosis, and advanced carpotarsal ossification [1.] The disease is extremely rare, with fewer than 110 reported cases to date. Two distinct types of DBQD have been classified based on the presence (type1) or absence (type 2) of specific hand abnormalities [2]. These features include a bifid distal thumb phalanx, an extra ossification center distal to the second metacarpal, and dislocation of the interphalangeal joints. The Kim variant, a milder variant of type 1, is characterized by short metacarpal joints, elongated phalanges, and advanced carpal bone age in the hand and spine [3]. DBQD type 1 is caused by a homozygous or compound heterozygous mutations in the CANT1 gene (chromosome 17q25) and DBQD type 2 results from mutations in the XYLT1 gene (chromosome 16p12).

Although skeletal findings are the most distinctive clinical features, multisystem involvement is also observed [4, 5]. Clinical manifestations include prenatal and postnatal disproportionate short stature, a short neck, multiple joint dislocations, brachydactyly (especially in the distal phalanges), progressive scoliosis, osteoporosis, progressive osteoarthritis, midline facial hypoplasia, prominent eyes, and a short nose. Additional systemic involvement includes pulmonary hypoplasia, horseshoe kidney, renal cyst, hydronephrosis, myopia, and glaucoma [6, 7].

CANT1 and xylosyltransferase 1 (XYLT1) genes are involved in the biosynthesis of glycosaminoglycans (GAGs), the main components of proteoglycans. In this process, CANT1 is responsible for the hydrolysis of UDP to UMP and phosphate in GAG chain synthesis, while XYLT1 is involved in the initiator and rate-limiting step of GAG biosynthesis. Mutations in genes encoding proteins associated with GAG synthesis lead to defects in the structure and function of proteoglycans. These defects in proteoglycan synthesis lead to diseases affecting connective, cartilage, and bone tissues. Cardiovascular structures are also affected by proteoglycan disorders due to their connective tissue content [8].

Aortic root dilatation and mitral valve prolapse, believed to be associated with defective proteoglycan production, have been described in this condition [9]. Some case reports also describe ventricular septal defects, atrial septal defects, and pulmonary stenosis [10–12]. The objective of our study was to provide insight into the cardiac involvement and clinical management of patients diagnosed with DBQD.

## Methods

The registry system at Hacettepe University İhsan Doğramacı Children's Hospital, Department of Pediatric Genetics, has recorded over 1500 patients diagnosed with skeletal dysplasia since 2005. Based on the updated 2023 Nosology classification of skeletal dysplasias, 11 patients diagnosed with Desbuquois dysplasia under the group of “Dysplasias with Multiple Joint Dislocations” (group 5) were identified. Among them, nine patients who agreed to participate and underwent cardiac evaluation at the Department of Pediatric Cardiology were included in the study. Data were retrospectively collected from hospital records. This study was designed as a single-center, descriptive, retrospective cohort study. Ethical approval was obtained from the Hacettepe University Health Sciences Research Ethics Committee (SBA 24/651), and the study adhered to the World Medical Association Declaration of Helsinki and Good Clinical Practice guidelines.

### Data collection

Demographic data of the patients, the presence of clinical symptoms and findings, involvement of other systems, clinical follow-up dates, genetic mutations, electrocardiogram interpretations, echocardiography findings and measurements, results from additional imaging modalities (if available), treatments, and council decisions (if available) were recorded.

### Cardiac assessment

Echocardiographic evaluations were performed using a Philips EPIQ CVx echocardiography device equipped with xMatrix transducer with 5 to 1 MHz extended operating frequency range for adult echo applications in 2D and Live xPlane modes. (Philips, Andover, MA, USA). M-mode echocardiographic recordings were analyzed, and *Z*-scores were calculated based on weight, height, and gender [13]. All of the metrics have been calculated based on the data obtained during the end-of-follow-up assessment. Mitral valve prolapse (MVP) is identified as a systolic displacement of one or both mitral leaflets of at least 2 mm above the plane of the mitral annulus in the sagittal echocardiographic view of the mitral valve [14, 15].

### Genetic analysis

Genomic DNA was extracted from peripheral blood lymphocytes using the QIAamp DNA Blood Mini Kit® (QIAGEN, N.V., the Netherlands), following the manufacturer’s instructions. Informed consent was obtained from all patients and their families. DNA sequencing was performed using the BigDye Terminator v3.1 Cycle Sequencing Kit, and sequencing products were analyzed using the ABI 3500 genetic analyzer (Thermo Fisher Scientific, Waltham, MA, USA). All exons and exon–intron boundaries of the CANT1 gene were sequenced. Sanger sequencing was performed to assess co-segregation among family members. The frequencies of identified variants were checked against multiple databases including NCBI dbSNP (build 141) (http://www.ncbi.nlm.nih.gov/SNP/), 1000 Genomes Project (http://www.1000genomes.org), and Genome Aggregation Database (gnomAD) (http://gnomad.broadinstitute.org/).

### Statistical analysis

Data analysis was performed using IBM SPSS version 23.0. Median (minimum–maximum) values were used to evaluate nonparametric variables, while percentages and ratios were utilized for expressing the frequency of categorical variables. *Z*-scores exceeding + 2 were considered pathological for ventricular and aortic dilatation.

## Results

A total of nine patients from eight unrelated families were included in the study, five of whom (55%) were female. The median age of the patients was 11 years (range 3.6–23.6 years), the median body weight was 15 kg (range 6–64 kg), and the median height was 94 cm (range 63–130 cm). The median age at first presentation to our clinic was 1.5 years (range 0.4–11.6 years), and the median follow-up period was calculated as 7.7 years (range 2.9–15.4 years). While seven patients remained asymptomatic during clinical follow-up, one experienced fatigue and another presented with tachypnea.

Patient 4 and patient 5 were siblings, while none of the other patients were related. Homozygous pathogenic variants in *CANT1* were identified in all patients. The two most common pathogenic variants were c.898 C > T (observed in 55% of patients) and c.739 T > C (observed in 33%). Additionally, one patient carried the pathogenic variant c.943 A > T (Table 1).

**Table 1:** Demographic, clinical, and genetic findings of the patients

Family no	Patients’ no	Age (y)	Gender	Weight (kg)	Weight (P/SDS)	Height (cm)	Height (P/SDS)	Cardiac symptom	Age at first admission	Follow-up period (y)	Homozygous CANT1 variant
1	1	3.6	M	7.5	< 0.02 p − 6.9 SDS	70	< 0.02 p − 7.5 SDS	None	8.5 mo	2.9	c.898 C > T; (p.Arg300 Cys) *(NM_001159773.2)
2	2	11.0	M	15	< 0.02 p − 5.4 SDS	96	< 0.02 p − 7.3 SDS	Fatigue	1.1 y	10.0	c.898 C > T; (p.Arg300 Cys) *(NM_138793.3)
3	3	9.6	F	11	< 0.02 p − 8.6 SDS	74	< 0.02 p − 10.6 SDS	None	4.5 y	5.2	c.898 C > T; (p.Arg300 Cys) *(rs267606701) (ENST00000302345.6)
4	4	13.7	F	30	< 0.02 p − 4.1 SDS	122	< 0.02 p − 6.3 SDS	None	1.4 y	12.3	c.739 T > C; (p.Tryp247 Arg) * (ENST00000302345.6)
4	5	11.1	F	25	1.16 p − 2.2SDS	116	< 0.02 p − 4.4 SDS	None	8.1 y	3.1	c.739 T > C; (p.Tryp247 Arg) * (ENST00000302345.6)
5	6	7.1	M	6	< 0.02 p − 15.8 SDS	63	< 0.02 p − 12.1 SDS	Tachypnea	1.5 y	5.6	c898 C > T; (p.Arg300 Cys) *(ENST00000302345.6)
6	7	9.6	F	9,5	< 0.02 p − 7.9 SDS	70	< 0.02 p − 10.5 SDS	None	1.9 y	7.7	c.943 A > T; (p.Lys315 Ter)** (ENST00000302345.6)
7	8	23.6	F	64	80 p 0.87 SDS	130	< 0.02 p − 5.6 SDS	None	11.6 y	12.1	c.739 T > C; (p.Trp247 Arg)*
8	9	15.8	M	25	< 0.02 p − 6.6 SDS	94	< 0.02 p − 11.8 SDS	None	4 mo	15.4	c.898 C > T; (p.Arg300 Cys) *(rs267606701) (ENST00000302345.6)

^*^Previously reported

^**^Novel

(*P* percentile, *SDS* standard deviation score)

The nonsense homozygous *CANT1* variant identified in Patient 7 (c.943 A > T; p.(Lys315 Ter), RefSeq: NM_138793.4) was evaluated for pathogenicity and causality according to the American College of Medical Genetics and Genomics (ACMG) Standards and Guidelines [16]. This variant is novel and has been classified as “likely pathogenic” based on the following criteria: PVS1 (a null variant in a gene where loss of function is a known disease mechanism) and PM2 (extremely low frequency in gnomAD population databases). Following the detection of this variant in the proband, Sanger sequencing was performed on both parents, revealing that they were heterozygous carriers. Patient 7, who carried this novel pathogenic variant, exhibited clinical findings such as dysmorphic facial features, short stature, pectus deformity, joint laxity and dislocation, and osteopenia. Radiological findings included a Swedish key proximal femur, monkey wrench appearance in the femoral heads, wide metaphyses, and advanced carpal-tarsal bone age, which were consistent with findings in other patients. The clinical and radiological findings of the patients are detailed in Table 2.

**Table 2:** Clinical and radiological findings of the patients

Patient’s no Cinical and radiological findings	1	2	3	4	5	6	7	8	9
Round face, prominent eyes, miyopia	+/±	+/+/+	+/+/ glaucoma	+/±	+/±	+/±	Midface hypoplasia/-/-	±/+	±/+
Short neck, pectus deformity	+/+	+/+	+/+	±	+/+	+/+	-/+	+/+	-/-
Joint laxity, dislocations, osteopenia	+/+/+	+/knee/+	+/knee/+	+/hip and knee/+	+/knee/+	±/+	+/knee/+	+/hip and knee/+	+/hip and knee/+
Coronal/sagittal cleft, scoliosis	sagittal cleft/+	-/+	-/+	-/+	-/+	sagittal cleft/+	Sagittal cleft/+	-/+	NA
Sweedish key proximal femur	+	+	+	Dysplastic femoral necks	+	+	+	-	NA
Monkey wrench appearance of the femoral heads	+	+	+	-	-?	+	+	-	NA
Wide metaphyses	+	+	+	+	-?	+	+	+	+
Brachydactyly, phalangeal dislocations	+/+	-/+	-/+	-/-	-/-	-/+	-/+	Shortness of 3rd and 4 th metacarp/-	First proximal phalanx/-
Radial/medial deviation of the fingers	+	+	+	-	-	+	+	-	Ulnar deviation
Delta/delta-like phalanx, accessory occification proximal phalanx, extra phalangial findings	-/-/bifid 2nd metacarp	-/-/-	+/±	-/-/-	-/-/-	±/-	-/-/-	-/-/short 3–4 th metacarp	+/+/short 2nd metacarp
Advanced carpal-tarsal bone age	+	+	+	+	-	+	+	+	+
Delayed motor development, hypotonia	+	+	+	-	+	+	+	+	±

### Cardiac findings and surgical considerations

Electrocardiographic evaluation revealed no significant arrhythmic changes, and Rhythm Holter monitoring was not performed, as none of the patients exhibited symptoms suggestive of arrhythmia. Echocardiographic M-mode assessment confirmed that all patients had normal cardiac systolic function. Echocardiographic measurement parameters, along with individualized *Z* scores, are provided in Table 3.

**Table 3:** Echocardiographic M mode and aortic measurements and evaluation of *Z* scores

Patients’ no	LVEDd (mm)/*Z* score	LVESd (mm)/*Z* score	LVEF (%)	LVFS (%)	IVSDd (mm)/*Z* score)	AV annulus (mm)/*Z* score)	SVS (mm)/*Z* score	STJ (mm)/*Z* score	Asc Ao (mm)/*Z* score
1	26.6/0.13	15.4/− 0.74	75	42	7.6/+ 3.54	13.2/3.8	20.3/4.2	14.9/3.8	17.5/3.2
2	51/7.6	36.4/7.9	55	29	12.4/7.6	22/5.2	27.5/5.12	27/7	30/7.25
3	26.8/− 0.93	14.7/− 2.03	78	45	5.4/0	16.8/3.46	22.3/4.8	14.1/1.9	18/3.2
4	36/− 1.3	22/− 1.37	68	37	5.7/− 1.43	17.3/1.32	24.7/1.78	19.7/1.52	21/1.1
5	36.8/− 0.37	23.4/− 0.32	67	36	6.5/− 0.35	15.6/0.71	23.2/1.72	17.7/0.92	18/0.16
6	32.5/3.38	15.5/− 0.32	84	52	6.4/2.05	19.3/7.31	26/6.8	19.7/6.31	18.5/5.1
7	24.6/− 1.45	13.1/− 2.6	80	47	6.7/1.94	12.6/2.45	17.8/2.72	15.4/3.52	19.7/5
8	41.2/− 1.8	24.3/− 1.95	72	41	7.3/− 1.2	19/1.3	25/0.6	18.7/− 0.4	24.5/2.3
9	43.3/5	24.5/3.07	75	43	7.3/3.7	23/6.71	34/7.55	29/7.83	34/8.5

*LVEDd* left ventricle end diastolic diameter, *LVESd* left ventricle end systolic diameter, *LVEF* left ventricle ejection fraction, *LVFS* left ventricular fractional shortening, *IVSDd* interventricular septum diastolic diameter, *AV* aortic valve, *SVS* sinus valsalva, *STJ* sinotubular junction, *Asc Ao* ascending aorta

Aneurysmal dilatation of the ascending aorta was observed in seven patients (77%), while aortic root dilatation was found in six patients (66%). The median individual *Z* scores for the sinus valsalva (SVS) in patients with aortic dilatation were 4.9 (range 2.7–7.5), while the median *Z* score in the ascending aorta was 5 (range 2.3–8.5).

Patient 2 had an SVS *Z* score of 5.1 and an ascending aorta *Z* score of 7.2. Aortic valve replacement and the Benthall procedure were recommended by the pediatric cardiology–cardiovascular surgery council, but the patient and family were hesitant due to surgical risks. Patient 9, with an SVS *Z* score of 7.2 and an ascending aorta *Z* score of 8.5, was deemed high risk for surgery due to comorbidities. Consequently, clinical follow-up was recommended. Patient 1 (8 months old) and patient 2 (13 months old) both exhibited aortic root and ascending aortic dilatation during their initial echocardiographic assessments. The first echocardiographic evaluation, performed at 8 months of age for patient 1 and 13 months of age for patient 2, also revealed dilation of the aortic root and ascending aorta.

Mitral valve prolapse was observed in seven patients (77%), and moderate mitral regurgitation accompanying it was seen in three patients on the evaluation of atrioventricular valves. A functional bicuspid aortic valve, resulting from fusion of the left and right coronary cusps, was identified in two patients (22%), with one exhibiting severe aortic insufficiency. Among the lesions with a left-to-right shunt, five patients (55%) had a small secundum atrial septal defect (ASD), one patient (11%) had a small perimembranous ventricular septal defect (VSD), and one patient (11%) had a coronary-cameral fistula (Table 4). During follow-up, ASD in two patients closed spontaneously. Additionally, the tricuspid apparatus created a pouch, leading to a spontaneous closure of the perimembranous VSD, as confirmed by color Doppler ultrasound, which detected no remaining shunt flow.

**Table 4:** Summary table of cardiovascular manifestations of patients

Homozygous CANT1 variant	Patients’ no	MVP	Mitral insufficiency	TVP	Bicuspid aorta	Aortic insufficiency	ASD	VSD	Left ventricular enlargement	Aortic root dilatation	Ascending aorta dilatation	Miscellaneous
c.898 C > T p.(Arg300 Cys)	1	+	Moderate	-	-	-	-	-	-	+	+	-
	2	+	-	+	Functional (left–right coronary cusp fusion)	Severe	Spontaneous closed	-	+	+	+	-
	3	+	Mild	+	-	-	-	-	-	+	+	-
	6	+	Moderate	+	-	Mild	Secundum	-	+	+	+	Coronary cameral fistula, severe pulmonary hypertension, biatrial dilatation
	9	+	Moderate	-	-	-	Secundum	-	+	+	+	-
c.739 T > C p.(Trp247 Arg)	4	+	Mild	+	-	-	-	-	-	-	-	Excessive trabeculation of the left ventricle
	5	+	Mild	+	-	-	-	-	-	-	-	-
	8	-	-	-	Functional (left–right coronary cusp fusion)	Mild	Spontaneous closed	-	-	-	+	-
c.943 A > T p.(Lys315 Ter)	7	-	Mild	-	-	-	Secundum	Spontaneous closed	-	+	+	-

*MVP* mitral valve prolapse, *TVP* tricuspid valve prolapse, *ASD* atrial septal defect, *VSD* ventricular septal defect

**Figure1:**
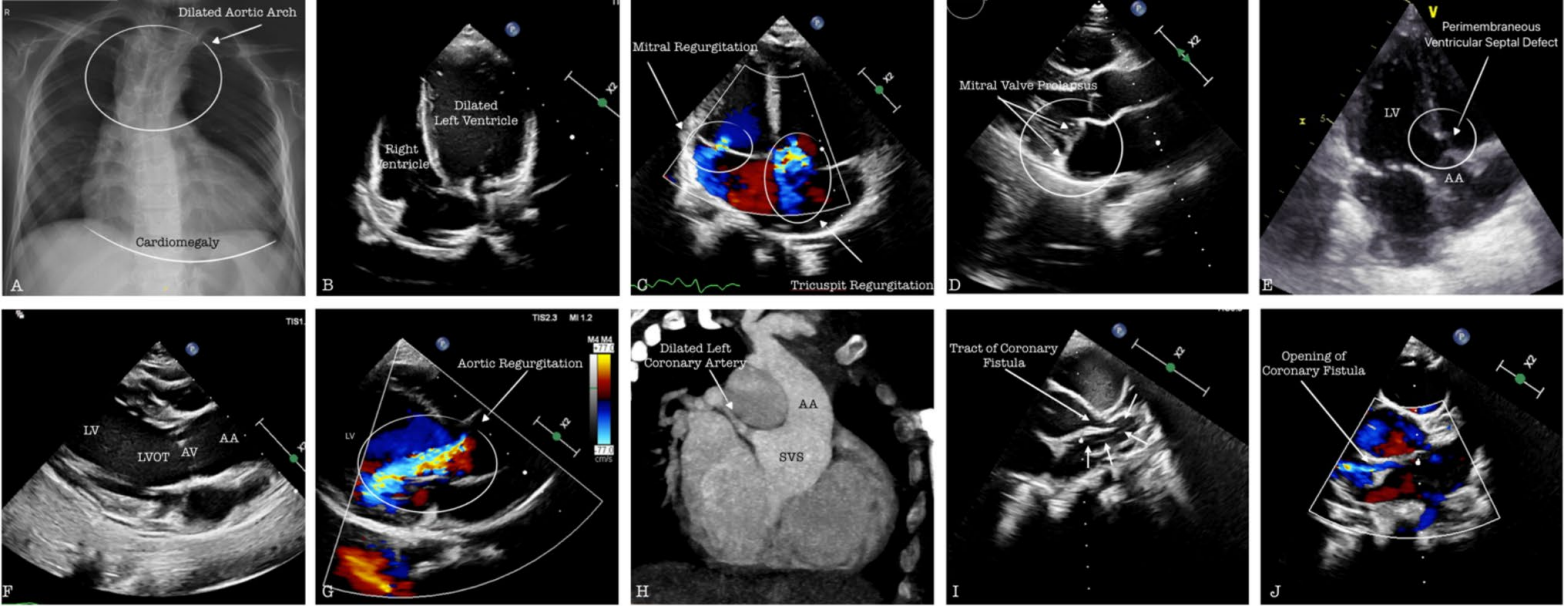
Collage of some screenshots from imaging methods. **A** Posterior-anterior view of chest X-ray: dilated aortic arch and cardiomegaly are noted. **B** Apical 4-chamber echocardiography image, left ventricular dilatation is seen. **C**–**D** Apical 4-chamber and long-axis echocardiography image: mitral valve prolapse and atrioventricular valve regurgitation are observed with color Doppler USG. **E** 2D apical 5-chamber echocardiography image, ventricular septal defect closed by spontaneous tricuspid pouch is observed. **F**–**G** Long-axis image shows left ventricle (LV), left ventricular outflow tract (LVOT), aortic valve (AV), dilated ascending aorta (AA), and aortic regurgitation. **H–J** 3D computed tomography and 2D short-axis echocardiographic images show a dilated sinus valsa (SVS) and ascending aorta (AA), coronary-cameral fistula originating from the left coronary artery and opening into the right atrium

In the cardiac evaluation of patient 6, enlargement of the right heart chambers was observed. A dilated left coronary artery and a fistula tract measuring up to 3.2 mm wide, opening into the right atrium from the distal left main coronary artery, were noted. The estimated right ventricular systolic pressure, measured from the moderate tricuspid insufficiency flow, was 80 mmHg, while the estimated mean pulmonary artery pressure, obtained from the pulmonary insufficiency flow, was 45 mmHg. The pulmonary artery width was 22 mm (+ 5.2 *Z* score). Computed tomography angiography confirmed the presence of a fistula between the circumflex artery and the right atrium. The extremely rare association of the syndrome and fistula presents numerous intriguing and unresolved questions. The patients have advanced skeletal and thoracic deformities, resulting in elevated pulmonary artery pressure related to pulmonary causes. Mortality reported in the DBQD patient group is mostly due to respiratory causes in early childhood [17]. Furthermore, pulmonary overflow with a left-to-right shunt occurs in the presence of a cardiac fistula. Echocardiography suggests that the shunt could be hemodynamically substantial and may lead to pulmonary hypertension. Catheterization and hemodynamic study are of great importance in clarifying the cause-effect relationship. Unfortunately, the patient’s family, who had initially planned for a transcatheter intervention, did not approve the procedure. Some diagnostic images of the patients are provided in Fig. 1.

## Discussion

The literature on DBQD is predominantly composed of case reports, as it is a rare form of chondrodysplasia. Consequently, it is difficult to determine its incidence [18]. Case reports generally emphasize the most prominent phenotypic feature—skeletal abnormalities. However, the presence of significant accompanying cardiac pathologies that may impact mortality has not been clearly elucidated. While the median age at first presentation is quite young due to the distinct phenotypic characteristics, our patient cohort spans the entire pediatric age range. Patients are typically diagnosed in infancy and, therefore, enter a prolonged phase of clinical follow-up.

Cardiac findings have been reported in a limited number of cases in the literature. In one case reported by Byrne et al., one case reported by Bloor et al., and a series of five patients reported by Baasanjav et al., a ventricular septal defect was detected in two patients [9–11]. Patent foramen ovale was detected in three patients, while atrial septal defect was detected in two patients. In our study, we observed spontaneous closure of VSD in one patient and spontaneous closure of ASD in two patients. Secundum ASD was observed in five of our cases. Notably, none of the described left-to-right shunt diseases were found to be hemodynamically significant.

Byrne et al. reported a homozygous missense variant (c.643G > A; p.(Glu215Lys), RefSeq: NM_138793.4) in one patient with concomitant cardiac abnormalities, including secundum ASD and a right-sided aortic arch, in addition to VSD. This patient succumbed to respiratory failure secondary to pneumonia at 6 months of age [11]. Regarding semilunar valve abnormalities, one patient in the literature exhibited pulmonary stenosis, while three patients had bicuspid aortic valves [10]. In our cohort, two patients had bicuspid aortic valves, but none exhibited aortic stenosis or pulmonary stenosis. However, one patient developed severe aortic insufficiency, likely attributable to bicuspid valve structure and aortic root dilatation.

Atrioventricular valve prolapse is the most frequently observed abnormality in DBQD. While four cases have been previously described in the literature, mild valve prolapse is a common feature in many patients [9]. In our study, seven out of nine patients exhibited mitral valve prolapse, while three patients had moderate mitral regurgitation, which could increase congestive cardiac load.

Coronary artery fistula, reported to have an incidence of 0.1% at coronary arteriography series [19], was observed in one of our patients who was previously reported [20]. The patient, diagnosed by echocardiography and confirmed by computed tomography, had accompanying pulmonary hypertension. Although the fistula was considered a potential cause of pulmonary hypertension, a hemodynamic study via cardiac catheterization was not performed due to the family’s concerns about the procedure and lack of consent.

Increased trabeculation and an increase in the non-compact segment were observed in the left ventricular myocardium of one patient. Despite preserved systolic function, the patient is being monitored long term for the development of cardiomyopathy. Currently, no cases of cardiomyopathy have been reported in the literature.

The most severe cardiac findings in our patients, affecting mortality and morbidity the most, are aortic root dilatation and ascending aortic aneurysm. Ascending aorta dilatation was observed in seven patients. Aortopathy has been previously described in four cases [9, 12]. In our study, two patients exhibited moderate aortic dilatation, while three had severe aortic dilatation, representing a critical aspect of the disease. Fortunately, no aortic dissections or mortalities occurred in our cohort. Although *Z* scores may be unreliable in patients with extreme height and weight, they remain valuable for monitoring disease progression and aortic diameter changes [21].

Although mortality has not yet been observed in our patients, previous studies indicate that mortality in DBQD type 1 patients is at least 33% within seven months follow-up time. Most fatal cases involve severe skeletal dysplasia and respiratory failure occurring in the neonatal and early infancy period [17]. However, data on long-term follow-up, aortic dissection risk, and overall mortality in these patients remain insufficient.

Heritable aortic diseases (HADs) encompass a diverse group of genetically transmitted disorders, with Marfan syndrome being the most extensively studied condition [22, 23]. The clinical management of DBQD patients has been modeled after the approach used for Marfan syndrome. According to the 2024 American Heart Association (AHA) guidelines on the cardiovascular management of aortopathy in children, our team has interpreted and applied the recommended clinical follow-up and treatment strategies for patients with DBQD, as summarized in Table 5 [24]. Notably, the use of beta-blockers and angiotensin receptor blockers in patients with Marfan syndrome has been shown to reduce the rate of aortic dilatation, with early initiation of treatment yielding the greatest benefit.

**Table 5:** Management of Desbuquois syndrome*

	Degree of aortopathy			
	None	Mild	Moderate	Severe
Age < 16 y (*Z* score)	< 2	≥ 2 and < 3.5	≥ 3.5 and < 5	≥ 5
Age ≥ 16 y (maximum dimension), cm	< 3.5	≥ 3.5 to < 4	≥ 4 to < 4.5	≥ 4.5
Clinical follow-up^1^	Every 12 months	Every 6 months	Every 3 months	Every 1–3 moths
Treatment	Consider single treatment	Single treatment^2^	Consider dual treatment	Dual treatment (if tolerated)^3^
Avoiding^4^	Agents that cause vasoconstriction, hypertension or tachycardia, including excessive caffeine use and long-term use of decongestants, fluoroquinolone antibiotic			
Activity	Encourage: Routine physical activity (30–60 min/d, 5 d/wk), physical education routine participation (peak exertion activities discouraged), recreational sports routine participation (with avoidance of contact sports) Discourage: Contact sports (e.g., tackle football, hockey, wrestling) and high-intensity training, leagues, and competitions			

^*^This table was interpreted by us for DBQD patients according to the recommendations using the Cardiovascular Management of Aortopathy in Children: A Scientific Statement from the American Heart Association 2024 guideline

^1^Monthly evaluation is recommended in case of increasing valve insufficiency, signs of congestive heart failure, rapid aortic dilatation (≥ 8 mm/year in the first 2 years, ≥ 5 mm/year thereafter, > 3 mm/year in adulthood)

^2^A: Beta-blocker: Propranolol before 4 years of age (non-selective, not preferred in chronic lung diseases); atenolol or metoprolol after 4 years of age (second generation) or B: Angiotensin receptor blocker: losartan for 6 months and above (creatinine, potassium, ALT, AST, and hemoglobin monitoring)

^3^The second treatment is added by titrating after the first treatment reaches the optimum level

^4^Both non-stimulant and stimulant medication in treating symptoms of attention-deficit/hyperactivity disorder should not be restricted for most children with aortopathy. Heart rate and blood pressure should be monitored closely

In this cohort, three different CANT1 variants were identified, one of which was novel. The patient with a novel homozygous pathogenic CANT1 variant and ventricular septal defect (VSD) (patient 7) exhibited dysmorphic features similar to those reported in other VSD cases in the literature. A postnatal skeletal survey revealed widened metaphyses, multiple joint dislocations, and a monkey-wrench appearance of the femoral heads in all affected individuals.

Studies on genotype–phenotype correlations in patients with DBQD are limited in the literature. In our cohort, we observed that certain genetic variants were associated with more severe clinical findings. Patients carrying the c.898 C > T, p.(Arg300 Cys) variant in the CANT1 gene (patients 1, 2, 3, 6, and 9) exhibited the most severe clinical manifestations, followed by patient 7 with the c.943 A > T, p.(Lys315 Ter) variant. Patients carrying the c.739 T > C, p.(Trp247 Arg) variant (patients 4, 5, and 8) showed comparatively milder but still significant symptoms.

Although the number of DBQD patients in our center is limited, these findings contribute valuable insights into genotype–phenotype correlations. Larger multicenter studies are necessary to further elucidate these relationships.

## Conclusion

DBQD is a rare form of chondrodysplasia associated with various cardiac abnormalities. The most frequently observed findings include aortic root and ascending aortic dilatation, as well as mitral valve prolapse, while atrial septal defect, ventricular septal defect, and bicuspid aortic valve occur less frequently. Aortopathy develops early and may progress to severe disease. Early detection of cardiac abnormalities and timely initiation of medical treatment can significantly improve long-term prognosis. Furthermore, identifying pathogenic variants in rare disorders within larger patient cohorts will contribute to a more comprehensive understanding of genotype-phenotype correlations.

## Data Availability

No datasets were generated or analysed during the current study.

## Abbreviations

ASD: Atrial septal defect
DBQD: Desbuquois dysplasia
SVS: Sinus valsalva
VSD: Ventricular septal defect
